# Integrating Regenerative Medicine and Stem Cell Therapy in Hearing Restoration: A Systematic Review

**DOI:** 10.7759/cureus.117530

**Published:** 2026-10-06

**Authors:** Isa D Hussain, Haidar Tanveer, Mohammad A Hussain, Hajra Rashid, Omnia Salah, Hisham Khalil

**Affiliations:** 1 Peninsula Medical School, University of Plymouth, Plymouth, GBR; 2 Gastroenterology, Hillingdon Hospitals NHS Foundation Trust, Uxbridge, GBR; 3 Urology, Luton and Dunstable Hospital, Luton, GBR; 4 Otolaryngology, University Hospitals Plymouth NHS Trust, Plymouth, GBR

**Keywords:** hearing restoration, inner ear regeneration, otolaryngology, regenerative medicine, sensorineural hearing loss, stem cell therapy

## Abstract

Hearing loss can adversely affect communication and language development and is associated with cognitive decline. Current treatments, including hearing aids and cochlear implants, can improve auditory function but do not regenerate damaged sensory cells. Stem-cell-based approaches have therefore attracted increasing interest as potential regenerative strategies, and stem cells have been used to generate inner-ear progenitor and hair-cell-like cells in vitro for hearing-loss research.

This systematic review aimed to evaluate the efficacy, safety, and regenerative potential of stem-cell-based therapies and related regenerative approaches relevant to hearing restoration. The included evidence spanned distinct levels of investigation: in vitro studies primarily informed regenerative and differentiation potential, animal studies evaluated selected auditory, cellular, and histological outcomes, and two small human studies provided preliminary clinical observations. Related genetic strategies were also included where directly relevant to auditory regeneration.

A literature search of PubMed and Google Scholar was conducted to identify relevant studies published between 2010 and 2025. Eligible studies investigated stem-cell-based therapies, stem/progenitor-cell approaches, or closely related regenerative strategies relevant to hearing restoration in human participants, animal models, or in vitro experimental systems. Approaches included mesenchymal stem cells (MSCs), induced pluripotent stem cells (iPSCs), embryonic stem cells (ESCs), related progenitor-cell strategies, and regenerative genetic approaches directly relevant to auditory restoration. Studies were required to report measurable auditory, cellular, histological, or functional outcomes relevant to inner-ear regeneration or hearing-related function.

Seventeen studies met the inclusion criteria. In vitro studies demonstrated differentiation of stem or progenitor cells toward inner-ear sensory and neural phenotypes, supporting regenerative potential but not clinical efficacy. Animal studies reported variable findings, including improvements or preservation in selected auditory measures such as auditory brainstem response (ABR) thresholds, alongside cellular and histological changes in some models; other studies demonstrated no significant functional improvement. The two small human studies reported no major treatment-related adverse events during their limited follow-up, while auditory outcomes were inconsistent. Substantial heterogeneity in cell sources, delivery methods, experimental models, and outcome measures limited direct comparison between studies.

Laboratory and animal studies support further investigation of stem-cell-based and related regenerative approaches for sensorineural hearing loss. However, clinical efficacy has not been established, and the available human evidence is limited to preliminary short-term observations from small studies. Standardized protocols and larger, long-term human studies are required to establish safety, durability, and clinical effectiveness.

## Introduction and background

Hearing loss is one of the most common sensory disorders worldwide. More than 1.5 billion people are estimated to have some degree of hearing loss, including approximately 403 million (26%) with moderate-to-complete hearing loss in the better-hearing ear [1,2]. Unaddressed hearing loss can adversely affect communication, educational development, social participation, and quality of life. In children, auditory deprivation may impair spoken-language development, while in older adults hearing loss is associated with cognitive decline [3,4].

Hearing loss is broadly classified as conductive, sensorineural, or mixed. Conductive hearing loss arises from abnormalities affecting sound transmission through the external or middle ear, whereas sensorineural hearing loss reflects pathology of the cochlea, auditory nerve, or central auditory pathways [5]. Once damaged, sensory structures within the mammalian inner ear have only limited capacity for spontaneous structural and functional regeneration, restricting the potential for biological recovery after severe injury [6].

Current rehabilitative treatments, including hearing aids and cochlear implants, can substantially improve auditory function in appropriately selected patients but do not regenerate lost cochlear sensory cells. Cochlear hair cells are specialized sensory cells within the inner ear that convert sound-induced mechanical vibrations into electrical signals that can be transmitted through the auditory nerve. Damage or loss of these cells is therefore an important cause of sensorineural hearing impairment. Consequently, regenerative strategies have attracted increasing interest as understanding of inner-ear development and repair has advanced [7,8].

Stem cells provide a potentially valuable platform for modeling the human inner ear and developing regenerative therapies because of their capacity for self-renewal and differentiation into specialized cell types. Pluripotent stem cells can generate derivatives of all three germ layers, making them particularly attractive for disease modeling, drug development, and regenerative research [9].

Stem cells can be classified according to their origin and differentiation potential. Embryonic stem cells (ESCs) are pluripotent cells derived from the inner cell mass of the blastocyst, whereas adult stem cells are tissue-resident populations with more restricted differentiation potential. Induced pluripotent stem cells (iPSCs), first generated by reprogramming differentiated adult cells, provide a pluripotent platform without requiring embryonic tissue. ESCs, iPSCs, and adult stem/progenitor cells have all been investigated in hearing-loss research, although each approach has distinct advantages and limitations relating to differentiation efficiency, immunogenicity, genomic stability, and clinical translation [10-12].

Although regenerative approaches to hearing loss have been investigated across in vitro systems, animal models, early human studies, and related genetic strategies, these different forms of evidence address distinct aspects of regenerative potential and cannot be interpreted as equivalent evidence of clinical hearing restoration. In vitro studies primarily evaluate cellular differentiation and mechanistic potential, animal studies permit assessment of selected auditory, cellular, and histological outcomes, and the limited human evidence provides preliminary clinical observations. This systematic review therefore aimed to synthesize the available evidence on the efficacy, safety, and regenerative potential of stem-cell-based and closely related regenerative approaches relevant to hearing restoration, while distinguishing clearly between laboratory, preclinical, and human evidence.

## Review

Materials and methods

This systematic review was conducted and reported in accordance with the Preferred Reporting Items for Systematic Reviews and Meta-Analyses (PRISMA) 2020 statement (Appendices) [13].

Protocol and Registration

No separate review protocol was prepared, and this systematic review was not prospectively registered.

Literature Search Strategy

A literature search of PubMed and Google Scholar was conducted to identify relevant studies published between 2010 and 2025. Boolean operators (“AND” and “OR”) were combined with terms relating to regenerative medicine, stem-cell therapy, otolaryngology, hearing loss, hearing restoration, and tissue regeneration. An example search strategy was: (“Regenerative Medicine” OR “Stem Cell Therapy”) AND (“Otolaryngology” OR “Hearing Restoration” OR “Tissue Regeneration”).

The exact final search date, complete source-specific search strategies, and precise number of Google Scholar results examined are not available from the retained review records. These limitations are acknowledged because they restrict full reproducibility of the search process. No additional databases, trial registries, or formal reference-list searches were documented in the retained records.

Inclusion and Exclusion Criteria

Studies were eligible if they investigated stem-cell-based therapy, stem/progenitor-cell approaches, or closely related regenerative strategies with direct relevance to hearing restoration. Eligible experimental settings included human participants, relevant animal models, and in vitro models of inner-ear development or regeneration. Interventions and approaches included mesenchymal stem cells (MSCs), iPSCs, ESCs, related progenitor-cell approaches, and regenerative genetic strategies directly relevant to auditory restoration. Studies were required to report measurable auditory, cellular, histological, or functional outcomes relevant to inner-ear regeneration or hearing restoration. English-language studies published between 2010 and 2025 and available in full text were considered. Requiring full-text availability may have influenced study inclusion and is considered a limitation of the review.

Studies were excluded if they focused on conditions unrelated to hearing loss or inner-ear regeneration, did not investigate a regenerative or cell-based approach relevant to auditory restoration, or did not provide sufficient primary outcome data. Conference abstracts, case reports, case series, editorials, commentaries, reviews without primary data, and studies with incomplete or ambiguous outcome data were also excluded. These criteria were applied to ensure that the review focused on primary evidence relevant to regenerative approaches for hearing restoration.

Study Selection

The study selection process is illustrated in Figure 1. A total of 5,849 records were identified through database searches: 842 from PubMed and 5,007 from Google Scholar. After removal of 4,012 duplicate records, 1,837 records underwent title and abstract screening, of which 952 were excluded. A total of 885 reports were recorded as having been sought for retrieval, with 674 recorded as not retrieved. The remaining 211 full-text reports were assessed for eligibility. Following full-text assessment, 17 studies met the eligibility criteria and were included in the systematic review.

**Figure 1:**
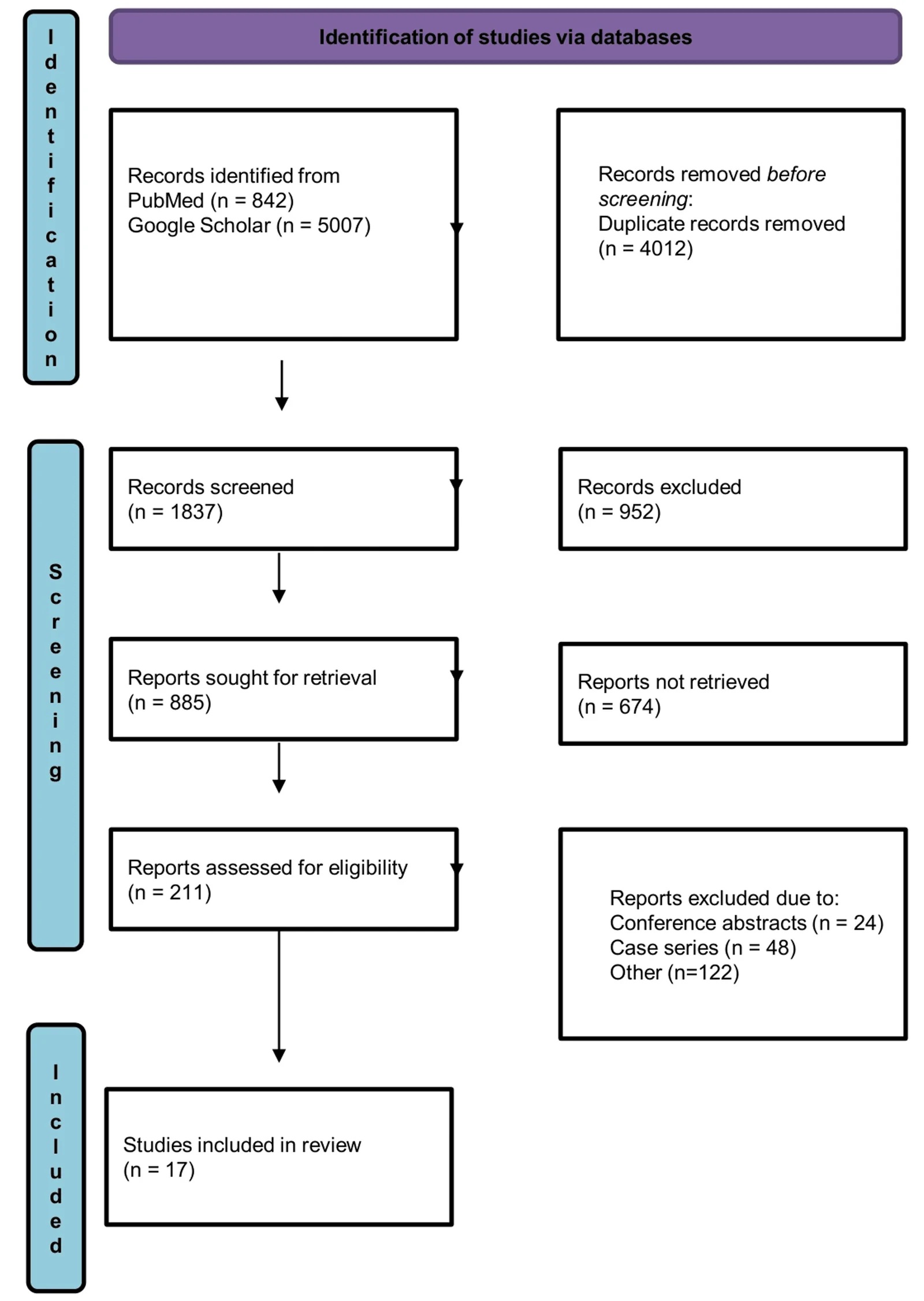
PRISMA flow chart showing selection criteria and inclusion of studies The study-selection figures are reported according to the retained review records. The number of reports recorded as not retrieved (674 of 885) is unusually high, and the original screening documentation was insufficient to determine whether all of these reports were genuinely unavailable or whether some may have represented exclusions or duplicate records at another stage of the selection process. The documented figures were therefore retained without retrospective reconstruction. PRISMA: Preferred Reporting Items for Systematic Reviews and Meta-Analyses

The retained review records do not document the exact method or software used to identify duplicate records across PubMed and Google Scholar. In addition, the number of reports recorded as not retrieved is unusually high. The available screening documentation was insufficient to reliably establish whether all 674 records were truly unavailable or whether some had been excluded or classified as duplicates at another stage of the selection process. Therefore, the reported figures were retained rather than retrospectively reconstructed. This limitation is acknowledged as potentially affecting confidence in the completeness and reproducibility of the study-selection process.

Data Extraction and Management

Data were extracted using a standardized form capturing author, year, study design, sample size, participant or experimental-model characteristics, stem-cell type, treatment or transplantation method, outcome measures, and reported effects on auditory function. The retained records do not document reviewer-specific procedures for data extraction or how any disagreements were resolved.

Because the included studies differed substantially in study design, experimental model, regenerative approach, delivery method, and outcome measurement, a quantitative meta-analysis was not considered appropriate. The findings were therefore synthesized narratively and organized according to evidence type, including in vitro studies, animal interventions, human studies, and related genetic strategies.

No formal risk-of-bias assessment, reporting-bias assessment, or certainty-of-evidence assessment was performed. These methodological limitations are acknowledged when interpreting the findings of the review.

Results

Seventeen studies met the predefined inclusion criteria. Seven were in vitro studies, eight were animal or other preclinical intervention studies, and two were human studies. The characteristics and principal findings of all included studies are summarized in Table 1.

**Table 1:** Characteristics and principal findings of studies included in the qualitative synthesis ABR: auditory brainstem response; DPOAE: distortion-product otoacoustic emission; OAE: otoacoustic emission; PTA: pure-tone audiometry; SNHL: sensorineural hearing loss; MSC: mesenchymal stem cell; iPSC: induced pluripotent stem cell

Study	Study design/model	Regenerative approach	Main outcomes assessed	Principal finding
Boddy et al. (2012) [14]	In vitro human study	Human bone-marrow-derived mesenchymal stem cells (MSCs)	Otic progenitor, hair-cell, and neural lineage differentiation	Conditioned culture induced otic progenitor markers followed by hair-cell and neuronal lineage markers, supporting the ability of human MSCs to differentiate toward inner-ear cell types.
Xu et al. (2016) [15]	Rat model of noise-induced hearing loss	Olfactory epithelium neural stem cells	ABR; transplanted-cell survival and migration	Cochlear implantation of neural stem cells improved noise-induced hearing impairment and demonstrated survival of transplanted cells within cochlear tissue.
Durán Alonso et al. (2012) [16]	In vitro human study	Bone-marrow-derived human MSCs	Hair-cell and auditory-neuronal differentiation	Human MSCs demonstrated the potential to differentiate toward inner-ear sensory hair-cell and auditory-neuronal phenotypes.
Lahlou et al. (2018) [17]	In vitro human study	Human induced pluripotent stem cells (hiPSCs)	Otic/placodal and sensory-lineage differentiation	FGF3/FGF10 promoted otic progenitor formation, while Notch-pathway modulation increased expression of otic sensory-lineage markers.
Ohnishi et al. (2015) [18]	In vitro human study	Human iPSCs	Hair-cell-like differentiation	A stepwise differentiation method generated hair-cell-like cells with hair-cell markers and stereocilia-like structures, although induction efficiency was limited.
Mattei et al. (2019) [19]	In vitro 3D vestibular-like organoid model	Human pluripotent stem cells	Inner-ear organoid development and vestibular-like hair-cell phenotype	Three-dimensional rotary culture generated inner-ear organoids containing enriched populations of hair cells with vestibular-like morphological and physiological characteristics. These findings support inner-ear developmental and regenerative modeling but do not constitute direct evidence of restored cochlear hearing.
Koehler et al. (2013) [20]	In vitro 3D culture study	Pluripotent stem cells	Sensory epithelium and functional hair-cell development	Three-dimensional differentiation generated inner-ear sensory epithelia containing mechanosensitive hair cells capable of forming connections with sensory neurons.
Gao et al. (2018) [21]	Mouse model of inherited hearing loss	Genetic intervention – in vivo genome editing targeting mutant TMC1; no cell transplantation	Auditory function and hearing preservation	Allele-targeted genome editing disrupted the pathogenic allele and reduced progressive hearing loss in the animal model. This finding is interpreted separately from the stem-cell transplantation studies.
Chen et al. (2012) [22]	Preclinical auditory-neuropathy model	Human embryonic-stem-cell-derived otic progenitors	Auditory-evoked responses; engraftment and neuronal differentiation	Otic neuroprogenitors engrafted and differentiated following transplantation and produced significant recovery of auditory-evoked response thresholds.
Baumgartner et al. (2018) [23]	Prospective human study; 11 children with acquired SNHL	Intravenous autologous umbilical cord blood	Safety; ABR and cochlear-nerve measures	No serious treatment-related adverse events were reported; five children showed reductions in ABR thresholds and four of these also showed improved cochlear-nerve latencies.
Lee et al. (2018) [24]	Human pilot study	Autologous bone-marrow-derived MSC transplantation	Safety; PTA, OAE, and ABR	No major treatment-related adverse events were reported during the study follow-up; no significant improvement in hearing was demonstrated.
Tang et al. (2016) [25]	Patient-derived in vitro study	iPSCs with CRISPR/Cas9 correction of MYO7A	Hair-cell morphology and electrophysiological function	Correction of the MYO7A mutation restored stereocilia-like organization and electrophysiological function in derived hair-cell-like cells.
Zhang et al. (2013) [26]	Rat model of spiral-ganglion-neuron degeneration	Olfactory-bulb-derived neural stem cells	Transplantation accuracy; migration and neuronal differentiation	Cochlear lateral-wall transplantation enabled neural stem cells to migrate efficiently into Rosenthal’s canal and differentiate into neurons.
Abd El Raouf et al. (2019) [27]	Guinea-pig model of carboplatin-induced hearing loss	Harderian-gland-derived stem cells	Auditory reflex measures; cochlear histology and morphometry	Stem-cell treatment was associated with reported improvements in Preyer pinna and startle auditory reflexes and in cochlear histological and morphometric measures, including hair-cell numbers, stria vascularis thickness, and spiral-ganglion cell counts. Animals were assessed for 35 days following stem-cell transplantation.
Kil et al. (2016) [28]	Animal model of SNHL	Human placenta-derived MSCs	ABR; DPOAE; spiral-ganglion regeneration	MSC transplantation improved ABR thresholds and DPOAE measures and increased spiral-ganglion neuron numbers, without evidence of acute immunological rejection during the study period.
Chang et al. (2021) [29]	Acute and chronic mouse neural hearing-loss models	Mouse embryonic stem cells (mESCs)	Cell survival/distribution and auditory neural signaling	Transplanted mESCs survived within the cochlea and increased auditory neural signaling toward the central auditory pathway, particularly in the chronic hearing-loss model.
Gökcan et al. (2016) [30]	Wistar rat model of ototoxic hearing loss	Mouse iPSC transplantation	ABR; histology and immunohistochemistry	No significant improvement in ABR thresholds was observed, and differentiated transplanted stem cells were not demonstrated histologically, illustrating that cell administration did not result in measurable auditory recovery in this model.

Two studies also investigated related genetic strategies: Tang et al. [25] used gene correction within a patient-derived in vitro iPSC model, while Gao et al. [21] investigated in vivo genome editing in an animal model. These genetic studies therefore overlap with the in vitro and animal categories rather than forming a separate mutually exclusive group.

In Vitro Models

In vitro studies primarily evaluated cellular differentiation, inner-ear developmental modeling, and regenerative potential. Human MSCs and pluripotent stem cells were differentiated toward otic progenitor, hair-cell-like, sensory, and neuronal phenotypes in several studies [14,16-20]. Three-dimensional culture approaches also generated inner-ear or vestibular-like organoid structures containing sensory-cell populations [19,20]. These findings support the biological and mechanistic potential of stem-cell-based approaches but do not establish clinical hearing restoration, efficacy, or safety in patients.

Animal Interventions

Animal studies assessed transplantation, cellular survival and migration, cochlear histology, neural signaling, and selected auditory outcomes. Some studies reported improvements or preservation in auditory measures, including auditory brainstem response (ABR) thresholds, together with histological or cellular changes [15,22,27,28]. Other studies produced more limited findings. Chang et al. principally demonstrated survival and distribution of transplanted cells and increased auditory neural signaling [29], while Gökcan et al. reported no significant improvement in ABR thresholds and did not demonstrate differentiated transplanted stem cells histologically [30]. These findings show that successful cell delivery or survival cannot be assumed to produce functional auditory recovery.

Human Studies

Two small human studies investigated mesenchymal or umbilical cord blood-derived cell approaches in patients with sensorineural hearing loss [23,24]. No major treatment-related adverse events were reported during the limited follow-up periods described in these studies. Auditory outcomes were inconsistent: Baumgartner et al. [23] reported reductions in ABR thresholds in some participants, whereas Lee et al. [24] did not demonstrate significant hearing improvement. These findings represent preliminary clinical observations and do not establish either long-term safety or clinical efficacy.

Related Genetic Strategies

Two included studies investigated genetic approaches relevant to auditory regeneration. Gao et al. used in vivo genome editing targeting mutant TMC1 in a mouse model and reported reduced progression of hearing loss [21]. This was a genetic intervention without cell transplantation and is therefore interpreted separately from the stem-cell transplantation studies. Tang et al. used CRISPR/Cas9 correction of an MYO7A-associated variant in patient-derived iPSCs and reported morphological and electrophysiological recovery in derived hair-cell-like cells [25]. This work provides mechanistic evidence in an in vitro model rather than clinical evidence of hearing restoration.

Heterogeneity

Considerable heterogeneity was present across the included studies in stem-cell source, experimental model, differentiation protocol, intervention and delivery method, study population, and outcome measurement. Auditory outcomes included measures such as ABR, distortion-product otoacoustic emission (DPOAE), otoacoustic emission (OAE), and pure-tone audiometry where applicable, while other studies focused primarily on cellular, histological, or electrophysiological outcomes. This heterogeneity limited direct comparison between studies and precluded meaningful quantitative pooling.

Discussion

Preclinical Evidence

The findings of this systematic review demonstrate that the evidence supporting regenerative approaches to hearing loss is predominantly derived from in vitro and animal studies. Mammalian cochlear sensory hair cells have limited natural regenerative capacity, which has driven interest in cell-based strategies capable of generating inner-ear sensory or neural phenotypes and potentially supporting damaged auditory structures [31].

In vitro studies demonstrated that human MSCs and pluripotent stem cells can be directed toward otic progenitor, sensory hair-cell-like, and auditory-neuronal phenotypes [14-20]. These findings support biological plausibility and provide useful platforms for studying inner-ear development, disease mechanisms, and candidate regenerative interventions. However, successful cellular differentiation in vitro does not establish clinical efficacy or safety in patients.

Animal studies provided additional evidence regarding cell survival, migration, cochlear integration, histological changes, neural signaling, and selected auditory outcomes. Some studies reported improvements or preservation in measures such as ABR thresholds or other auditory measures following regenerative interventions [15,22,27,28]. These findings suggest that selected experimental approaches may influence auditory function in preclinical models.

However, the animal evidence was not uniformly positive. Chang et al. principally examined the distribution and survival of transplanted mouse ESCs and reported increased auditory neural signaling toward the central auditory pathway, particularly in the chronic neural hearing-loss model [29]. In contrast, Gökcan et al. reported no significant improvement in ABR thresholds and did not demonstrate differentiated transplanted stem cells histologically [30]. This negative finding is important because it illustrates that successful cell administration or persistence cannot be assumed to produce functional auditory recovery.

Clinical Evidence

Human evidence remains limited. Two small studies evaluated mesenchymal or umbilical cord blood-derived cell approaches in patients with sensorineural hearing loss [23,24]. No major treatment-related adverse events were reported during the limited follow-up periods described in these studies, providing preliminary short-term tolerability information only. The small sample sizes and limited duration of observation do not establish general or long-term safety.

Functional auditory outcomes were also inconsistent. Baumgartner et al. reported reductions in ABR thresholds in a proportion of pediatric participants, while Lee et al. did not demonstrate significant hearing improvement following autologous bone-marrow-derived mesenchymal stem-cell transplantation [23,24]. These findings remain preliminary and do not establish clinical efficacy.

Related Genetic Strategies

Related genetic approaches may complement cell-based regenerative strategies, particularly for genetically mediated hearing loss. Gao et al. used in vivo genome editing targeting mutant TMC1 in a mouse model and reported reduced progression of hearing loss [21]. This intervention did not involve cell transplantation and should therefore be interpreted separately from the stem-cell transplantation studies.

Tang et al. [25] combined patient-derived iPSC technology with CRISPR/Cas9 correction of an MYO7A-associated variant and demonstrated morphological and electrophysiological recovery in derived hair-cell-like cells. This study provides mechanistic evidence relevant to personalized regenerative approaches but remains an in vitro model and does not establish clinical hearing restoration in patients.

Translational Challenges and Outcome Measures

A major challenge for clinical translation is the ability of transplanted or regenerated cells to survive, integrate into appropriate cochlear structures, differentiate into the required cellular phenotype, and contribute to durable auditory function. Experimental studies have explored direct cochlear delivery, including transplantation through the cochlear lateral wall to target regions containing spiral ganglion neurons [26]. Although local delivery may improve targeting, invasive cochlear procedures may also carry a risk of additional auditory injury [32].

The included studies also used heterogeneous outcome measures. Animal and human investigations assessed auditory function using measures including ABRs, DPOAEs, OAEs, and pure-tone audiometry where applicable, whereas several laboratory studies focused primarily on cellular, histological, morphological, or electrophysiological outcomes. These outcomes should not be interpreted as equivalent measures of hearing restoration.

The heterogeneity of study designs, experimental models, regenerative approaches, delivery techniques, and outcome measures prevented meaningful quantitative pooling. The evidence was therefore synthesized narratively rather than by meta-analysis.

Limitations

This review has several important methodological limitations. No separate review protocol was prepared, and the review was not prospectively registered. The retained search documentation is incomplete: the exact final search date, complete source-specific search strategies, and precise number of Google Scholar results examined are unavailable. The search was limited to PubMed and Google Scholar, and no additional databases, trial registries, or formal reference-list searches were documented. Relevant studies indexed elsewhere may therefore have been missed.

Full-text availability was also an eligibility requirement and may have influenced which studies were included. In addition, 674 of 885 reports sought for retrieval were recorded as not retrieved. This is an unusually high proportion. The original screening documentation is insufficient to determine reliably whether all of these reports were genuinely unavailable or whether some may have represented exclusions or duplicate records at another stage of the review process. The documented figure was therefore retained, but this uncertainty reduces confidence in the completeness of the study-selection process.

The exact duplicate-identification procedure across PubMed and Google Scholar was not documented in the retained records, and reviewer-specific procedures for study selection and data extraction were also not separately recorded. No formal risk-of-bias assessment, reporting-bias assessment, or certainty-of-evidence assessment was performed. Consequently, the methodological quality and certainty of the included evidence cannot be quantified formally, and the findings should be interpreted cautiously.

Finally, the evidence base itself was highly heterogeneous and predominantly preclinical. Only two small human studies were included, with limited follow-up and inconsistent auditory outcomes. The current evidence therefore cannot establish the clinical efficacy or long-term safety of stem-cell-based approaches for hearing restoration.

Future Directions

Future research should prioritize standardization of stem-cell source, cell characterization, differentiation protocols, dose, delivery route, diagnostic criteria, and auditory outcome measures. Demonstration of long-term cellular stability, appropriate integration, and clinically meaningful functional benefit will be essential before routine clinical translation.

Large, well-designed human studies with longer follow-up are required to determine the durability of any auditory benefit and to identify patient groups most likely to respond. Future studies should also use prospectively defined outcomes, transparent reporting, formal risk-of-bias assessment, and reproducible search and analysis methods. Carefully controlled combinations of cell therapy with gene editing, biomaterials, or pharmacological modulation may also warrant further investigation.

## Conclusions

The available evidence suggests that stem-cell-based and related regenerative approaches have biological and preclinical potential for sensorineural hearing loss. In vitro studies demonstrate the capacity of stem and progenitor cells to differentiate toward inner-ear sensory and neural phenotypes, while animal studies provide evidence of variable effects on selected auditory, cellular, and histological outcomes. However, these findings do not establish clinical hearing restoration.

Human evidence remains limited to two small studies that provide preliminary short-term observations regarding tolerability and variable auditory outcomes. Clinical efficacy and long-term safety have therefore not been established. Substantial heterogeneity in regenerative approaches, study designs, experimental models, delivery techniques, and outcome measures, together with important methodological limitations of the review, further limits the strength of the conclusions that can be drawn.

Future research should prioritize standardized protocols, rigorous methodological assessment, and larger, well-designed human studies with longer follow-up to determine safety, durability, and clinical effectiveness.

## Appendices

**Table 2:** PRISMA 2020 checklist for reporting of this systematic review PRISMA: Preferred Reporting Items for Systematic Reviews and Meta-Analyses

PRISMA item	Reporting information	Location
1 - Title	Systematic review identified in the title.	Title
2 - Abstract	Summary of background, objective, methods, results, and conclusion provided.	Abstract
3 - Rationale	Rationale for conducting the review described.	Introduction & Background
4 - Objectives	Review objective stated.	Abstract
5 - Eligibility criteria	Inclusion and exclusion criteria described.	Materials and methods - Inclusion and Exclusion Criteria
6 - Information sources	PubMed and Google Scholar and the 2010–2025 publication eligibility period are reported. The exact final search date is not available from the retained review records. No additional databases, trial registries, or formal reference-list searches were documented.	Materials and methods - Literature Search Strategy; Discussion - Limitations
7 - Search strategy	The principal Boolean search terms are reported. Complete source-specific search strategies and the precise number of Google Scholar results examined are not available from the retained records, limiting full reproducibility.	Materials and methods - Literature Search Strategy; Discussion - Limitations
8 - Selection process	Study-selection stages and numbers are reported. The exact duplicate-identification method and reviewer-specific screening procedure were not documented in the retained records.	Materials and methods - Study Selection; Figure 1; Discussion - Limitations
9 - Data collection	A standardized data-extraction form was used. Reviewer-specific extraction procedures and methods for resolving disagreements were not documented in the retained records.	Materials and methods - Data Extraction and Management; Discussion - Limitations
10a - Outcomes	Auditory, cellular, histological, and functional outcomes relevant to inner-ear regeneration or hearing restoration were sought, including measures such as ABR, OAEs, and pure-tone audiometry where applicable.	Materials and methods - Inclusion and Exclusion Criteria; Data Extraction and Management
10b - Other variables	Author, year, study design, sample size, model/population, stem-cell type, intervention, and outcomes were extracted.	Materials and methods - Data Extraction and Management
11 - Risk of bias	No formal risk-of-bias assessment was performed. This is acknowledged as a methodological limitation affecting confidence in the findings.	Materials and methods - Data Extraction and Management; Discussion - Limitations
12 - Effect measures	No quantitative meta-analysis was performed because substantial heterogeneity in study design, experimental model, intervention, delivery method, and outcome measurement precluded meaningful pooling. Findings were synthesized narratively.	Materials and methods - Data Extraction and Management; Results - Heterogeneity; Discussion
13a - Studies contributing to synthesis	Determined using the predefined eligibility criteria.	Inclusion and Exclusion Criteria; Data Extraction and Management
13b - Data preparation	No additional data transformations were reported.	Not performed/reported
13c - Presentation	Results of individual studies were presented narratively and summarized in Table 1.	Results; Table 1
13d - Synthesis	Eligible studies were synthesized narratively and organized according to evidence type: in vitro models, animal interventions, human studies, and related genetic strategies.	Materials and methods - Data Extraction and Management; Results
13e - Heterogeneity	Heterogeneity was assessed qualitatively across study design, experimental model, regenerative approach, delivery method, population, and outcome measurement. No formal statistical heterogeneity analysis was performed.	Results - Heterogeneity; Discussion
13f - Sensitivity analysis	Not applicable; no sensitivity analyses were performed.	Not applicable
14 - Reporting bias	No formal reporting-bias assessment was performed. This is acknowledged as a methodological limitation.	Materials and methods - Data Extraction and Management; Discussion - Limitations
15 - Certainty assessment	No formal certainty-of-evidence assessment was performed. This is acknowledged as a methodological limitation.	Materials and methods - Data Extraction and Management; Discussion - Limitations
16a - Study selection	Search and selection results are reported with a PRISMA flow diagram.	Study Selection; Figure 1
16b - Excluded studies	Grouped reasons for full-text exclusion are shown in Figure 1; individual excluded studies are not separately cited.	Figure 1
17 - Study characteristics	The characteristics and principal findings of the included studies are presented in Table 1.	Table 1
18 - Risk of bias in studies	No formal study-level risk-of-bias assessment was performed; therefore, methodological quality could not be formally quantified.	Discussion - Limitations
19 - Individual study results	Principal outcomes and findings of the included studies are summarized in Table 1 and discussed narratively.	Results; Table 1; Discussion
20a - Results of synthesis	Seventeen studies were synthesized narratively according to evidence type. Seven were in vitro studies, eight were animal/preclinical intervention studies, and two were human studies; two genetic-strategy studies overlapped with the in vitro and animal categories.	Results; Table 1
20b - Statistical synthesis	Not applicable; no meta-analysis was performed.	Not applicable
20c - Heterogeneity investigations	Not applicable; no formal subgroup or meta-regression analyses were performed.	Not applicable
20d - Sensitivity analyses	Not applicable.	Not applicable
21 - Reporting biases	No formal reporting-bias assessment was performed.	Materials and methods - Data Extraction and Management; Discussion - Limitations
22 - Certainty of evidence	No formal certainty-of-evidence assessment was performed; this limits confidence in the overall conclusions.	Materials and methods - Data Extraction and Management; Discussion - Limitations
23a - Interpretation	Findings interpreted in relation to the existing evidence.	Discussion
23b - Limitations of evidence	Limitations include heterogeneity of study designs and outcomes, predominantly preclinical evidence, only two small human studies, limited follow-up, and inconsistent auditory outcomes.	Discussion - Limitations; Conclusions
23c - Limitations of review process	Limitations include absence of a separate protocol, incomplete search documentation, unavailable exact final search date, use of only PubMed and Google Scholar, full-text availability requirements, unusually high numbers of reports recorded as not retrieved, undocumented duplicate-handling procedures, and absence of formal risk-of-bias, reporting-bias, and certainty assessments.	Discussion - Limitations
23d - Implications	Implications for future research and clinical translation discussed.	Discussion; Conclusions
24a - Registration	The review was not prospectively registered.	Materials and methods - Protocol and Registration
24b - Protocol	No separate review protocol was prepared.	Materials and methods - Protocol and Registration
24c - Amendments	Not applicable because no prospective registration or separate protocol was available.	Not applicable
25 - Support	No financial or non-financial support was received for this review.	Not applicable
26 - Competing interests	No competing interests declared.	Cureus Conflicts of Interest declaration
27 - Availability of materials	Data-extraction materials are not currently available.	Not available
